# The complete mitochondrial genome of *Ogmocotyle ailuri*: gene content, composition and rearrangement and phylogenetic implications

**DOI:** 10.1017/S0031182023000379

**Published:** 2023-07

**Authors:** Jun-Feng Gao, Ai-Hui Zhang, Wei Wei, Bin Jia, Jun Zhang, Ben Li, Ying-Yu Chen, Yun-Yi Sun, Mei-Ru Hou, Xue-Wei Liu, Jia-Wen Wang, Xin-Hui Zhang, Chun-Ren Wang

**Affiliations:** 1 Key Laboratory of Bovine Disease Control in Northeast China, Ministry of Agriculture and Rural affairs; Heilongjiang Provincial Key Laboratory of Prevention and Control of Bovine Diseases; College of Animal Science and Veterinary Medicine, Heilongjiang Bayi Agricultural University, Daqing 163319, China; 2 Branch of Animal Husbandry and Veterinary of Heilongjiang Academy of Agricultural Sciences, Qiqihar, China

**Keywords:** Gene arrangement, mitochondrial genome, molecular phylogeny, *Ogmocotyle ailuri*

## Abstract

Trematodes of the genus *Ogmocotyle* are intestinal flukes that can infect a variety of definitive hosts, resulting in significant economic losses worldwide. However, there are few studies on molecular data of these trematodes. In this study, the mitochondrial (mt) genome of *Ogmocotyle ailuri* isolated from red panda (*Ailurus fulgens*) was determined and compared with those from Pronocephalata to investigate the mt genome content, genetic distance, gene rearrangements and phylogeny. The complete mt genome of *O. ailuri* is a typical closed circular molecule of 14 642 base pairs, comprising 12 protein-coding genes (PCGs), 22 transfer RNA genes, 2 ribosomal RNA genes and 2 non-coding regions. All genes are transcribed in the same direction. In addition, 23 intergenic spacers and 2 locations with gene overlaps were determined. Sequence identities and sliding window analysis indicated that *cox*1 is the most conserved gene among 12 PCGs in *O. ailuri* mt genome. The sequenced mt genomes of the 48 Plagiorchiida trematodes showed 5 types of gene arrangement based on all mt genome genes, with the gene arrangement of *O. ailuri* being type I. Phylogenetic analysis using concatenated amino acid sequences of 12 PCGs revealed that *O. ailuri* was closer to *Ogmocotyle sikae* than to *Notocotylus intestinalis*. These data enhance the *Ogmocotyle* mt genome database and provide molecular resources for further studies of Pronocephalata taxonomy, population genetics and systematics.

## Graphical abstract

**Figure:**
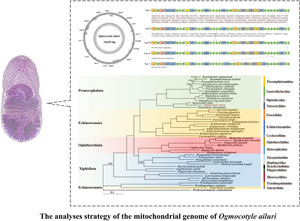

## Introduction

The red panda (*Ailurus fulgens*) is a globally threatened species with an estimated >40% population decline over the past 50 years in the wild. It occurs in the wild in India, Nepal, Myanmar and China, where it is mainly distributed in Sichuan, Yunnan, Guizhou and Tibet, with numbers estimated to be 3000–7000 (Wei *et al*., 1999). The International Union for Conservation of Nature upgraded the status of the species from vulnerable to endangered in 2015 (Goździewska-Harłajczuk *et al*., 2021). The red panda was also included in a national key protected wildlife list in China by the State Forestry and Grassland Administration and the Ministry of Agriculture and Rural Affairs in 2021 (www.forestry.gov.cn/main/5461/20210205/122418860831352.html).

Disease is one of the most important factors to consider when managing endangered species (Sharma and Achhami, 2022). Parasitic diseases are particularly serious given that they can influence the reproductive rate and population abundance in captive wild animals (Albon *et al*., 2002). *Ogmocotyle* spp. are intestinal trematodes of various mammalian species and belong to the family Notocotylidae, suborder Pronocephalata, order Plagiorchiida. *Ogmocotyle* trematodes have been reported in a variety of definitive hosts, including ruminants (Ma *et al*., 2016), primates (Coil, 1966; Yoshimura *et al*., 1969; Iwaki *et al*., 2012), waterfowl (Xu *et al*., 2021), panda (Li *et al*., 2020) and red panda (Pice, 1954). The flukes mainly inhabit the small intestine, with large numbers of flukes resulting in ogmocotylosis, which manifests clinically as diarrhoea, mucosal haemorrhage, haemorrhagic enteritis, malnutrition and even death (Ma *et al*., 2016). Trematodes of this genus are mainly distributed in Asian countries, including China, Korea, Japan and India (Coil, 1966; Eom *et al*., 1984; Iwaki *et al*., 2012; Ma *et al*., 2016).

Morphological descriptions have had a key role in identifying and differentiating parasites, with some synonym species within the genus *Ogmocotyle* from various hosts being reported. Sey and Graber (1991) conducted a systematic morphological description of *Ogmocotyle* trematodes known at that time, and identified 6 valid species in the genus: *Ogmocotyle africanum*, *Ogmocotyle ratti*, *Ogmocotyle fujianensis*, *Ogmocotyle indica* (synonym *Ogmocotyle capricori*), *Ogmocotyle sikae* (synonym *Ogmocotyle pygargi*) and *Ogmocotyle ailuri* (synonym *Ogmocotyle macacae*) (Sey and Graber, 1991; Yang and Zhang, 2013). Two species, *O. indica* and *O. ailuri*, were reported to infest red pandas. The main morphological identification characteristics distinguishing the 2 species were the location of the cirrus pouch and testis shape. In *O. indica*, the cirrus pouch occurs longitudinally in one-third of the body, and the testis is either kidney shaped or oval with a smooth surface; by contrast, in *O. ailuri*, the cirrus pouch lies on the front half of the body in an arc shape, and the testis is lobular (Sey and Graber, 1991).

The emergence of molecular data has resolved many of the limitations associated with morphological approaches for comparing related species and any further in-depth analyses. For example, genus *Orientobilharzia* was originally called *Schistosoma*, and then re-named *Ornithobilharzia* based upon the presence of numerous testes. Subsequently, it transferred to the new genus *Orientobilharzia* to accommodate blood flukes whose definitive hosts were mammals (Wang *et al*., 2009). Aldhoun and Littlewood (2012) then proposed on molecular grounds that the genus *Orientobilharzia* was in fact a junior synonym of *Schistosoma* (Aldhoun and Littlewood, 2012). In addition, controversy has surrounded whether the hookworms *Bunostomum trigonocephalum* and *Bunostomum phlebotomum* represent different species or strains, given significant similarities in morphology. However, molecular data were used to show that *B. trigonocephalum* and *B. phlebotomum* represent distinct but closely related species (Gao *et al*., 2014). Thus, more molecular data are needed to reassess traditional morphological classifications.

As a result of maternal inheritance, an apparent lack of recombination, rapid evolutionary rates and comparatively conserved genomic structures, mitochondrial (mt) genomes are highly suitable for trematode taxonomy, population genetics and systematics studies (Ichikawa-Seki *et al*., 2017; Le *et al*., 2020; Valentyne *et al*., 2020). Although 6 valid species have been recognized in the genus *Ogmocotyle*, only the *O. sikae* mt genome is currently available in GenBank. The paucity of molecular data is a limitation for population genetic and phylogenetic studies of these parasites.

Therefore, the objectives of the current study were to determine the complete *O. ailuri* mt genome, compare the mtDNA genome with those previously reported for Pronocephalata trematodes, and reconstruct the phylogenetic relationships with other trematodes based on a dataset comprising the concatenation of 12 protein-coding gene (PCG) nucleotide sequences. The results provide a molecular resource for further studies of Pronocephalata taxonomy, population genetics and systematics.

## Materials and methods

### Parasites and total genomic DNA isolation

An autopsy was performed to determine the cause of death in red pandas in Longsha Zoological and Botanical Garden, Heilongjiang Province, China. Trematodes were collected from the small intestine of red panda following Wildlife Protection Law protocols of the People's Republic of China (Fang *et al*., 2022). The trematodes were identified to the species level based on previously described morphological keys (Sey and Graber, 1991) (Fig. S1). They were then fixed in 75% ethanol and stored at −20°C until use. Total genomic DNA was isolated from a single fluke using a sodium dodecyl sulphate/proteinase K treatment, followed by spin-column purification using Genomic DNA Purification System (Promega, Madison, Wisconsin, USA) according to the manufacturer's protocol.

### Sequencing and mt genome assembly

Illumina paired-end shotgun libraries were prepared using the standard protocol of the Nextera^™^ DNA Sample Prep Kit (Epicentre, Madison, Wisconsin, USA) and sequenced using an Illumina NovaSeq sequencing platform (Personal Biotechnology Co, Ltd, Shanghai, China) using 2 × 100 cycles. Raw sequence data were deposited into the Short Read Archive database (www.ncbi.nlm.nih.gov/sra/) under accession number PRJNA896356. Clean data without sequencing adapters were assembled using NOVOPlasty software (Dierckxsens *et al*., 2017) with the parameters of the genome range (13 000–15 000) and k-mer 39. The completeness of the mt genome assembly was further verified by polymerase chain reaction and Sanger sequencing using 4 pairs of primers designed on the basis of conserved regions (Table S1; Fig. S2).

### Sequence analysis and gene annotation

Mt genome sequences were used for a BLAST search of the NCBI database (http://blast.ncbi.nlm.nih.gov/Blast) (Altschul *et al*., 1997). Twelve PCGs were initially identified using ‘ORF Finder’ through NCBI and the MITOS Web Server (Rombel *et al*., 2002) to specify the mt genetic code of invertebrates. The MITOS Web Server was then used to calculate the potential stem-loop secondary structures within these tRNA gene sequences (Bernt *et al*., 2013). The codon usage of the 12 PCGs was analysed using the invertebrate genetic code and the Codon Usage web server (www.bioinformatics.org/sms2/codon_usage.html). An analysis of compositional skews was conducted using equations (1) and (2) (Perna and Kocher, 1995):1
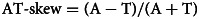
2
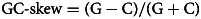
The relative synonymous codon usage of the 12 PCGs was calculated using the Sequence Manipulation Suite (www.detaibio.com/sms2/codon_usage.html) (Stothard, 2000). A sliding window analysis [window length = 300 base pairs (bp), step size = 10 bp] was conducted using DnaSP v.5 (Librado and Rozas, 2009) to assess nucleotide diversity Pi (*π*) between 12 PCGs from 14 Pronocephalata mt genomes. Nucleotide diversity was plotted against the mid-point positions of each window, and gene boundaries were identified. The differences between nucleotide and amino acid sequences were calculated using MEGA 11.0 and MegAlign (Burland, 2000; Tamura *et al*., 2021). A gene map of the mt genome was constructed using the online mitochondrial visualization tool OrganellarGenomeDRAW (Lohse *et al*., 2013). To determine the occurrence of *Plagiorchiida* spp. gene recombination, the gene arrangement of the *O. ailuri* mt genome was compared with those of 47 Plagiorchiida species currently available. The circular mt genomes were linearized at the 5′ end of their *cox*3 genes in the H-strand direction, as previously reported (Gao *et al*., 2021).

### Phylogenetic analysis

Phylogenetic relationships among the 48 representative members of *Plagiorchiida* available in GenBank (Table S2) were determined based on concatenated amino acid sequences of 12 PCGs, using *Postharmostomum commutatum* (NC_044643) as the outgroup. The PCG amino acid sequences were aligned using MAFFT v7.471 (Katoh and Standley, 2013), and the alignment was processed for elimination of poorly aligned sites and divergent regions using the GBlocks Server (http://molevol.cmima.csic.es/castresana/Gblocks_server.html).

Phylogenetic trees were reconstructed using Bayesian inference (BI) methods. The best-fit substitution model for phylogenetic analysis of the amino acid alignment was determined using jModeltest under Akaike information criterion, and identified as the SYM + I + G model (Darriba *et al*., 2012). MrBayes v3.2.6 (Huelsenbeck and Ronquist, 2001) was used to conduct BI analysis. This analysis was performed for 10 000 000 generations, in 2 simultaneous runs, with 4 chains (3 heated and 1 cold), to catalyse swapping among the Markov-chain Monte Carlo chains. Trees were sampled every 1000 generations. Tracer v1.6 software (http://tree.bio.ed.ac.uk/software/tracer/) was used to investigate the convergence of sampled parameters and potential autocorrelation (effective sample size for all parameters >200). In addition, the average standard deviations of the split frequencies were checked between both runs (<0.01). Bayesian posterior probabilities were obtained from the 50% majority-rule consensus of the post-burn-in trees sampled at stationarity after removing the first 25% of trees as a ‘burn-in’ stage. The final phylogenetic tree was graphically visualized and edited using FigTree v1.4.3 (http://tree.bio.ed.ac.uk/software/figtree/).

## Results

### General features of the *O. ailuri* mt genome

**Figure 1.**
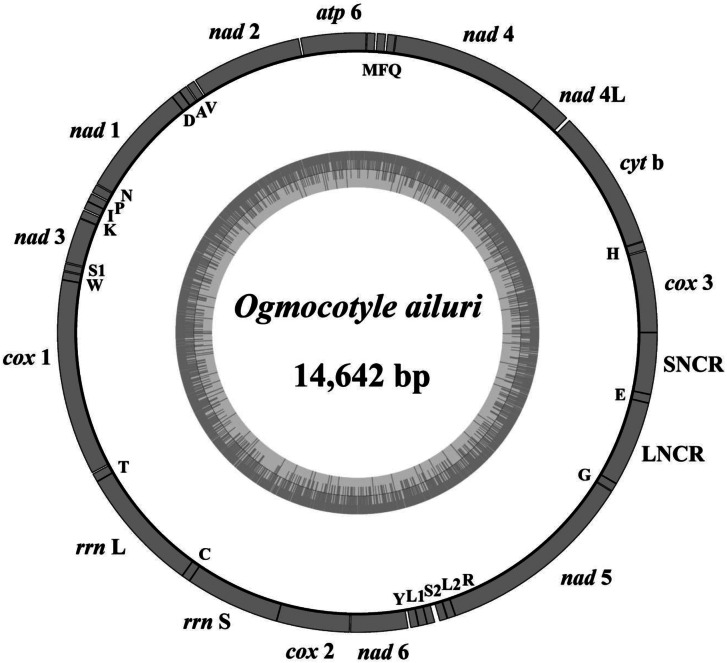
Gene map of the mt genome of *Ogmocotyle ailuri*. All 22 tRNAs are designated by the 1-letter code for the corresponding amino acid, with numerals differentiating each of the 2 leucine- and serine-specifying tRNAs (L1 and L2 for codon families CUN and UUR, respectively; S1 and S2 for codon families UCN and AGN, respectively), LNCR and SNCR refer to long and short non-coding region. All genes are transcribed in the anticlockwise direction.

**Figure 2.**
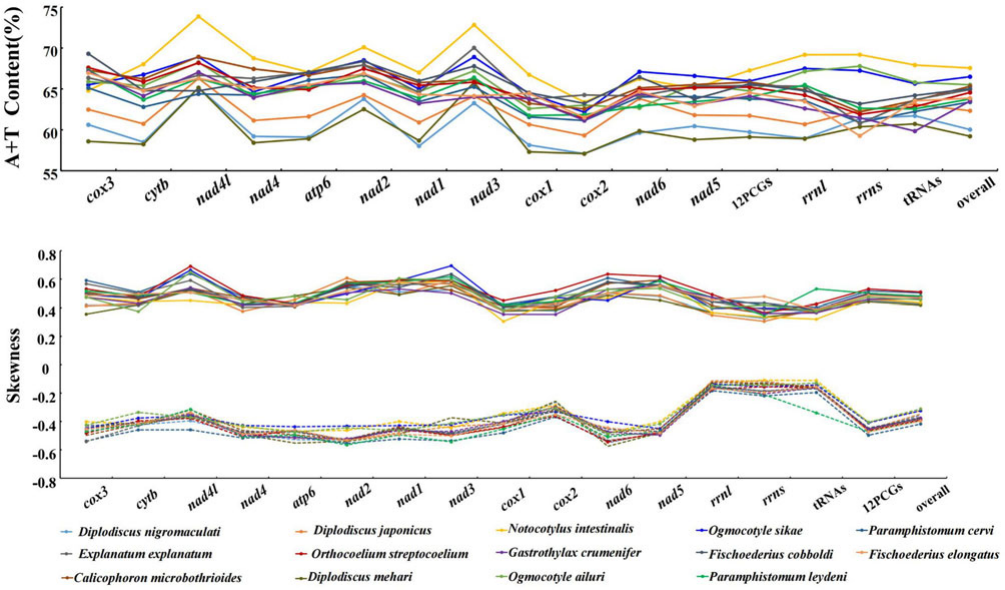
A + T content and nucleotide skew of genes, individual elements and the complete mt genome of 14 Pronocephalata trematodes. Lines colour and point represent a specific Pronocephalata species.

The *O. ailuri* mt genome (GenBank accession number OP414758) was a typical closed circular molecule, 14 642 bp in size. It contained 12 PCGs (*cox*1–3, *nad*1–6, *nad*4L, *cyt*b and *atp*6), 22 tRNA genes (1 for each amino acid and 2 each for leucine and serine), 2 rRNA genes (*rrnL* and *rrnS*) and 2 non-coding regions [NCRs; long (LNCR) and short (SNCR)], but lacked *atp*8 (Fig. 1; Table 1). All the genes were transcribed in the same direction. There were 23 intergenic spacers, ranging from 1 to 37 bp, and 2 locations with gene overlaps, ranging from 1 to 40 bp (Table 1). The nucleotide composition of the entire mt genome of *O. ailuri* was biased towards A and T, with an overall AT content of 65.51% (Fig. 2A).

**Table 1.** Features of the mt genome of *Ogmocotyle ailuri*

Genes	Location	Length (bp)	Start codons/stop codons	Anti-codons	Intergenic spacers (bp)
*cox*3	1–645	645	ATG/TAG		7
*trn*H	653–719	67		GTG	3
*cyt*b	723–1832	1110	ATG/TAA		26
*nad*4L	1859–2110	252	ATA/TAG		−40
*nad*4	2071–3357	1287	ATG-TAG		4
*trn*Q	3362–3424	63		TTG	14
*trn*F	3439–3506	68		GAA	14
*trn*M	3521–3586	66		CAT	4
*atp*6	3591–4106	516	ATG/TAG		14
*nad*2	4121–4990	870	ATG/TAG		12
*trn*V	5003–5067	65		TAC	8
*trn*A	5076–5140	65		TGC	1
*trn*D	5142–5211	70		GTC	0
*nad*1	5212–6111	900	ATG/TAA		6
*trn*N	6118–6187	70		GTT	9
*trn*P	6197–6265	69		TGG	0
*trn*I	6266–6330	65		GTA	10
*trn*K	6341–6407	67		CTT	3
*nad*3	6411–6770	360	ATG/TAG		4
*trn*S1	6777–6836	60		GCT	5
*trn*W	6842–6907	66		TCA	0
*cox*1	6908–8455	1548	ATA/TAA		9
*trn*T	8465–8533	69		TGT	0
*rrn*L	8534–9527	995			0
*trn*C	9528–9596	69		GCA	0
*rrn*S	9597–10 344	748			0
*cox*2	10 345–10 920	576	ATG/TAG		6
*nad*6	10 927–11 379	453	ATG/TAG		18
*trn*Y	11 398–11 461	64		GTA	0
*trn*L1	11 462–11 529	68		TAG	−1
*trn*S2	11 529–11 591	63		TGA	37
*trn*L2	11 629–11 691	63		TAA	2
*trn*R	11 694–11 758	65		TCG	0
*nad*5	11 759–13 339	1581	GTG/TAG		4
*trn*G	13 344–13 410	67		TCC	0
SNCR	13 411–14 087	677			0
*trn*E	14 088–14 155	68		TTC	0
LNCR	14 156–14 642	487			0

In the *O. ailuri* mt genome, the 12 PCGs accounted for 10 098 bp and encoded 3354 amino acids, excluding the termination codons. The average AT content of the 12 PCGs was 64.8% (Fig. 2A). The initiation and termination codons of the PCGs in the *O. ailuri* mt genome are listed in Table 1. The most common initiation codon for *O. ailuri* was ATG (9 of 12 PCGs), followed by ATA (2 of 12 PCGs) and GTG (1 of 12 PCGs). Two types of termination codon, TAG (9) and TAA (3), occurred in the *O. ailuri* mt genome. The codon usage analyses of the 12 PCGs in the mt genome are summarized in Table 2. The most frequent amino acid was Phe (TTT, 10.46%), followed by Leu (TTG, 9.25%), Val (GTT, 5.94%) and Leu (TTA, 5.44%). The least frequently used were Leu (CTC, 0.06%), Thr (ACC, 0.12%) and Arg (CGC, 0.15%) (Table 2).

**Table 2.** Codon usage analysis of 12 PCGs in the mitochondrial genome of *Ogmocotyle ailuri*

Codon	Number	/1000	Fraction	Codon	Number	/1000	Fraction
GCG(Ala)	26	7.74	0.23	CCG(Pro)	25	7.44	0.3
GCA(Ala)	21	6.25	0.18	CCA(Pro)	15	4.47	0.18
GCU(Ala)	60	17.86	0.52	CCU(Pro)	37	11.02	0.45
GCC(Ala)	8	2.38	0.07	CCC(Pro)	6	1.79	0.07
UGU(Cys)	103	30.66	0.89	CAG(Gln)	18	5.36	0.6
UGC(Cys)	13	3.87	0.11	CAA(Gln)	12	3.57	0.4
GAU(Asp)	56	16.67	0.85	CGG(Arg)	6	1.79	0.1
GAC(Asp)	10	2.98	0.15	CGA(Arg)	14	4.17	0.23
GAG(Glu)	57	16.97	0.7	CGU(Arg)	35	10.42	0.58
GAA(Glu)	24	7.14	0.3	CGC(Arg)	5	1.49	0.08
UUU(Phe)	351	104.5	0.96	AGG(Ser)	49	14.59	0.13
UUC(Phe)	14	4.17	0.04	AGA(Ser)	38	11.31	0.1
GGG(Gly)	97	28.88	0.36	AGU(Ser)	93	27.69	0.25
GGA(Gly)	34	10.12	0.13	AGC(Ser)	12	3.57	0.03
GGU(Gly)	122	36.32	0.45	UCG(Ser)	31	9.23	0.08
GGC(Gly)	17	5.06	0.06	UCA(Ser)	34	10.12	0.09
CAU(His)	47	13.99	0.85	UCU(Ser)	95	28.28	0.26
CAC(His)	8	2.38	0.15	UCC(Ser)	14	4.17	0.04
AUU(Ile)	128	38.11	0.91	ACG(Thr)	20	5.95	0.25
AUC(Ile)	12	3.57	0.09	ACA(Thr)	12	3.57	0.15
AAG(Lys)	53	15.78	0.64	ACU(Thr)	43	12.8	0.54
AAA(Lys)	30	8.93	0.36	ACC(Thr)	4	1.19	0.05
UUG(Leu)	244	72.64	0.48	GUG(Val)	122	36.32	0.3
UUA(Leu)	182	54.18	0.36	GUA(Val)	76	22.63	0.19
CUG(Leu)	14	4.17	0.03	GUU(Val)	199	59.24	0.49
CUA(Leu)	26	7.74	0.05	GUC(Val)	12	3.57	0.03
CUU(Leu)	44	13.1	0.09	UGG(Trp)	61	18.16	0.58
CUC(Leu)	2	0.6	0	UGA(Trp)	45	13.4	0.42
AUG(Met)	100	29.77	0.55	UAU(Tyr)	144	42.87	0.85
AUA(Met)	82	24.41	0.45	UAC(Tyr)	26	7.74	0.15
AAU(Asn)	52	15.48	0.88	UAG(*)	9	2.68	0.75
AAC(Asn)	7	2.08	0.12	UAA(*)	3	0.89	0.25

The 22 tRNA genes identified in the *O. ailuri* mt genome ranged from 60 to 70 bp. The total length of the 22 tRNA genes was 1457 bp, and the AT content was 65.80%. *Rrn*L was located between *trn*T and *trn*C, and *rrn*S between *trn*C and *cox*2. *rrn*L and *rrn*S were 995 and 748 bp in *O. ailuri*, respectively, and had an AT content of 67.14 and 67.78%, respectively. The mt genome of *O. ailuri* contained 2 NCRs, LNCR located between *trn*G and *trn*E, and SNCR located between *trn*E and *cox*3. LNCR and SNCR were 677 and 487 bp in *O. ailuri*, respectively, and had an AT content of 69.40 and 66.47%, respectively.

### Comparative analysis of *O. ailuri* mt genome within Pronocephalata trematodes

The complete mt genome of *O. ailuri* was relatively large among Pronocephalata species, although was shorter than that of *Diplodiscus nigromaculati* (14 697 bp) and *Gastrothylax crumenifer* (14 801 bp), but longer than that of other Pronocephalata (Table 3). The gene transcription direction of the complete mt genomes of *O. ailuri* was the same as all Pronocephalata mt genomes reported thus far. The rank order of the 12 PCGs of *O. ailuri* by length was as follows: *nad*5 *>* *cox*1 *>* *nad*4 *>* *cyt*b *>* *nad*1 *>* *nad*2 *>* *cox*3 *>* *cox*2 *>* *atp*6 *>* *nad*6 *>* *nad*3 *>* *nad*4L.

**Table 3.** Comparison of 12 PCGs among *Ogmocotyle ailuri* and other suborder Pronocephalata trematodes

Genes	Dn	Dm	Dj	Ni	Ogs	Oga	Pc	Cm	Ee	Ors	Gc	Fc	Fe	Pl	Nucleotides similarity (%)	Amino acid similarity (%)
	No. nucleotides/No. amino acid															
*cox*3	645/214	645/214	645/214	645/214	645/214	645/214	645/214	645/214	645/214	645/214	645/214	645/214	645/214	645/214	53.0–89.5	47.2–95.8
*cyt*b	1116/371	1116/371	1113/370	1089/362	1110/369	1110/369	1113/310	1116/371	1113/370	1113/370	1113/370	1113/370	1113/370	1113/370	71.7–91.2	73.7–97.0
*nad*4l	264/87	264/87	264/87	264/87	267/88	252/83	264/87	264/87	264/87	264/87	264/87	264/87	264/87	264/87	68.9–91.3	58.6–98.9
*nad*4	1287/428	1287/428	1287/428	1287/428	1287/428	1287/428	1281/426	1281/426	1281/426	1281/426	1281/426	1281/426	1281/426	1281/426	65.3–90.0	62.2–95.1
*atp*6	516/171	516/171	516/171	516/171	516/171	516/171	516/171	516/171	516/171	516/171	516/171	516/171	516/171	516/171	67.8–92.6	60.2–98.8
*nad*2	870/289	870/289	870/289	870/289	870/289	870/289	873/290	873/290	876/291	858/285	858/285	873/290	876/291	873/290	62.9–90.0	54.0–92.4
*nad*1	903/300	903/300	903/300	891/296	891/296	900/299	897/298	897/298	897/298	897/298	903/300	897/298	897/298	897/298	70.5–91.0	68.2–97.0
*nad*3	354/117	354/117	354/117	357/118	357/118	360/119	357/118	357/118	357/118	356/118	378/125	357/118	357/118	357/118	65.2–88.5	50.4–95.8
*cox*1	1548/515	1572/523	1548/515	1548/515	1557/518	1548/515	1545/514	1542/513	1542/513	1542/513	1542/513	1542/513	1542/513	1545/514	72.4–92.5	74.3–97.3
*cox*2	585/194	585/194	585/194	579/192	579/192	576/191	579/192	585/194	582/193	582/193	582/193	582/193	582/191	582/193	67.0–94.0	63.5–97.9
*nad*6	456/151	456/151	456/151	453/150	453/150	453/150	453/150	453/150	453/150	453/150	453/150	453/150	501/166	453/150	63.4–89.3	56.7–95.4
*nad*5	1563/520	1563/520	1563/520	1581/526	1581/526	1560/519	1581/526	1581/526	1581/526	1581/526	1581/526	1578/525	1581/526	1584/527	64.5–89.0	57.3–94.9
Total nt/aa	10 107/3357	10 131/3365	10 104/3356	10 080/3348	10 113/3359	10 077/3347	10 104/3296	10 110/3358	10 110/3357	10 088/3351	10 116/3360	10 047/3355	10 155/3373	10 110/3358	68.2–90.3	51.5–95.3
Total size(bp)	14 697	14 210	14 179	14 317	14 307	14 642	14 014	14 028	13 968	13 800	14 801	14 256	14 120	14 050	64.3–89.8	

*Note:* Dn, *Diplodiscus nigromaculati*; Dm, *Diplodiscus mehari*; Dj, *Diplodiscus japonicus*; Ni, *Notocotylus intestinalis*; Ogs, *Ogmocotyle sikae*; Oga, *Ogmocotyle ailuri*; Pc, *Paramphistomum cervi*; Cm, *Calicophoron microbothrioides*; Ee, *Explanatum explanatum*; Ors, *Orthocoelium streptocoelium*; Gc, *Gastrothylax crumenifer*; Fc, *Fischoederius cobboldi*; Fe, *Fischoederius elongatus*; Pl, *Paramphistomum leydeni*.

The A + T content of *O. ailuri* mt genomes was lower than those of *O. sikae* and *Notocotylus intestinalis*, which was the lowest in the Notocotylidae reported thus far (Fig. 2A). The AT bias of the *O. ailuri* mt genome was consistent with other Pronocephalata. The *O. ailuri* AT-skew values were negative, and the GC-skew values were positive, ranging from −0.486 (*nad*6) to −0.144 (*rrn*L) and 0.375 (*cyt*b) to 0.604 (*nad*1), respectively (Fig. 2B).

**Fig. 3.**
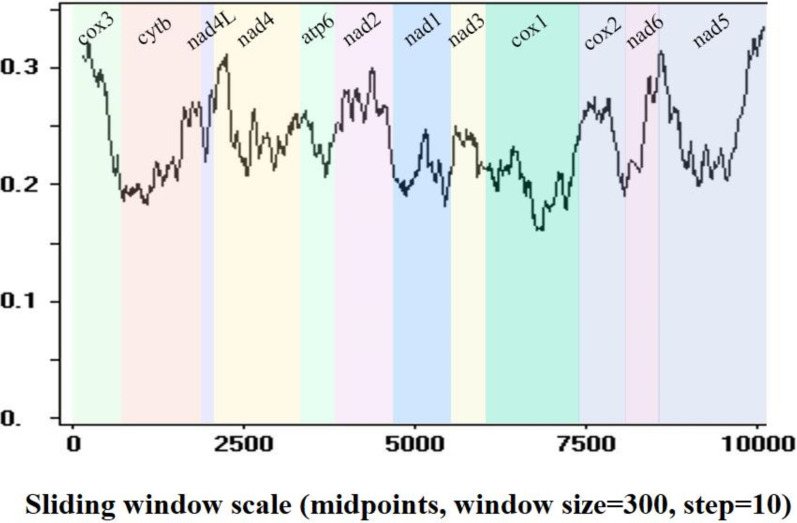
Sliding window analysis of the complete mt genome sequences of 14 Pronocephalata trematodes. A sliding window of 300 bp (in 10 bp overlapping steps) was used to estimate nucleotide diversity Pi (*π*) across the alignments. Nucleotide diversity was plotted against the mid-point positions of each window. Each gene boundary is identified.

Sequence identities and sliding window analyses were used to determine the diversity and mutation rate of mt genes among 14 Pronocephalata. Sequence identities of the 12 PCGs from the 14 Pronocephalata species were 68.2–90.3% at the nucleotide level and 51.5–95.3% at the amino acid level. The complete mt genome nucleotide identities among the 14 trematodes ranged from 64.3 to 89.8% (Table 3). Among the 12 PCGs, *cox*3 had the fewest similarities among the 14 species, whereas *cox*1 had the highest similarity (Table 3). A sliding window analysis of the concatenated nucleotide sequences of 12 PCGs showed obvious differences in 12 PCGs from the 14 Pronocephalata trematodes. By computing the number of variable positions per unit length of gene, the curve indicated that *cox*1 (0.161) was the least variable gene, whereas *cox*3 (0.321) and *nad*5 (0.336) showed the highest sequence variation (Fig. 3).

### Gene arrangements

**Figure 4.**
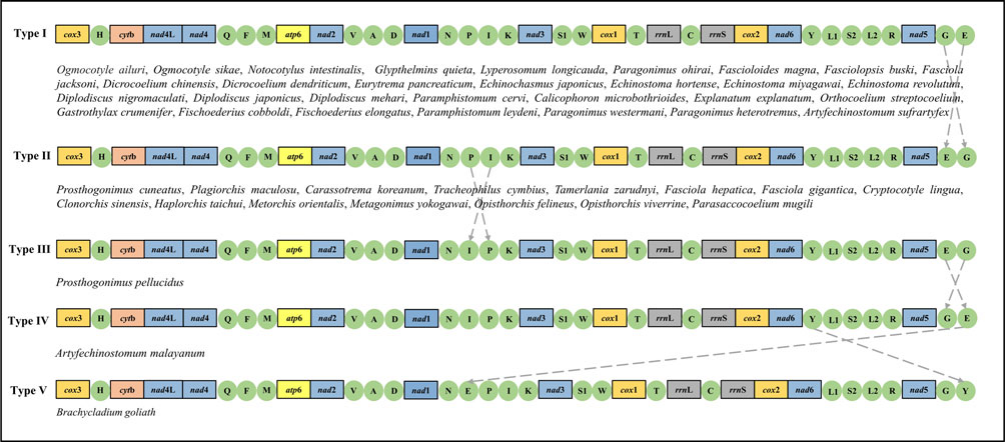
Mt genome arrangement in *Ogmocotyle ailuri* compared with those in Plagiorchiida trematodes. The circular mt genomes were linearized at the 5′ end of *cox*3 gene for illustration purpose. Non-coding regions were not shown.

The sequenced mt genome of the 48 Plagiorchiida showed 5 types of gene arrangement, with that of the *O. ailuri* mt genome being type I (Fig. 4). The gene arrangements of Plagiorchiida shared an identical arrangement based on 12 PCGs and 2 rRNA genes (the order: *cox*3 > *cyt*b > *nad*4L > *nad*4 > *atp*6 > *nad*2 > *nad*1 > *nad*3 > *cox*1 > *rrn*L > *rrn*S > *cox*2 > *nad*6 > *nad*5), and the gene rearrangement events only occurred in transposed tRNAs.

The rearrangement events mainly occurred in 2 gene blocks (*trn*N-*trn*P-*trn*I-*trn*K and *trn*G-*trn*E) of the mt genome in Plagiorchiida. Compared with type I rearrangements, *trn*G and *trn*E were interchanged within *nad*5 and *cox*3 in type II rearrangements. Compared with type II rearrangements, *trn*P and *trn*I were interchanged with *nad*1 and *nad*3 in type III rearrangements. Compared with type III rearrangements, *trn*E and *trn*G were interchanged with *nad*5 and *cox*3 in type IV rearrangements. *trn*E between *nad*5 and *cox*3 in type IV rearrangements was translocated between *nad*1 and *nad*3 in type V rearrangements. Additionally, *trn*Y, which is between *nad*6 and *nad*5 in type IV rearrangements, was translocated between *nad*5 and *cox*3 in type V rearrangements.

### Phylogenetic analysis

**Figure 5.**
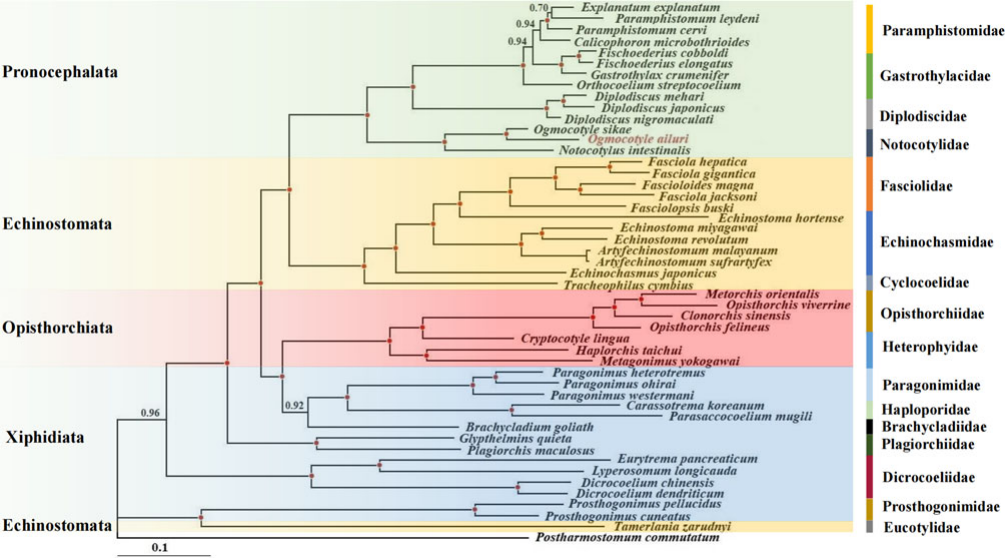
Phylogenetic relationships of *Ogmocotyle ailuri* with other 48 Plagiorchiida trematodes based on concatenated amino acid sequences of 12 protein-coding genes analysed by BI using *Postharmostomum commutatum* as the outgroup. Posterior probability values are indicated. Suborders and families are highlighted by individual colours. Circles indicate BI = 1.0, other values are given above the nodes.

BI approaches were used to estimate the phylogenetic position of *O. ailuri* within 47 other Plagiorchiida trematodes based on the concatenated amino acid sequences of 12 PCGs (Fig. 5). The result showed that the Pronocephalata and Opisthorchiata suborders form independent monophyletic groups in the phylogenetic tree. Xiphidiata trematodes have a complicated taxonomic relationship, although each family clustered together in 1 branch; however, Xiphidiata trematodes formed 4 lineages rather than a monophyletic group. *P*rosthogonimus *pellucidus* and *P*rosthogonimus *cuneatus* clustered together with *Tamerlania zarudnyi* to form a group on the outermost branch, which was next to other Plagiorchiida trematodes. Except for *T. zarudnyi* (Eucotylidae), 12 Echinostomata trematodes clustered together to form a group.

The branch of Pronocephalata includes the clades Paramphistomidae, Gastrothylacidae, Diplodiscidae and Notocotylidae. Paramphistomidae, Gastrothylacidae and Diplodiscidae clustered together to form a group, with Notocotylidae forming another group. In the Notocotylidae branch, *O. ailuri* isolated from this study was closer to *O. sikae* than to *N. intestinalis*.

## Discussion

In this study, the entire mt genome of *O. ailuri* was sequenced for the first time. The mt genome of *O. ailuri* was the classical structure of trematodes, contained 12 PCGs, 22 tRNA genes, 2 rRNA genes and 2 non-coding regions. There were a 40 bp gene overlap between *nad*4L and *nad*4 in *O. ailuri* mt genome, the result was consistent with most Plagiorchiida, except *Dicrocoelium dendriticum* (8 bp), *Dicrocoelium chinensis* (8 bp), *Lyperosomum longicauda* (37 bp), *Paragonimus westermani* (14 bp) and *N. intestinalis* (0 bp) (Biswal *et al*., 2014; Liu *et al*., 2014; Suleman *et al*., 2020; Xu *et al*., 2021).

A common feature of the mt genome in most metazoans is a bias towards a higher representation of A and T nucleotides, which leads to subsequent biases in the corresponding encoded amino acids (Hu *et al*., 2020). The AT content of the entire mt genome and the 12 PCGs was 65.51 and 64.8%, respectively, and the result was consistent with other Plagiorchiida trematodes biased towards A and T (Atopkin *et al*., 2021; Ivashko *et al*., 2022). The negative AT-skew indicated a higher incidence of T compared with A nucleotides, and the positive GC-skew indicated that G was more abundant than C (Le *et al*., 2019). The AT-skew values were negative, and the GC-skew values were positive in *O. ailuri* mt genome; these trends in AT- and GC-skew were similar to those of other Pronocephalata (Yan *et al*., 2013; Xu *et al*., 2021).

Three types of initiation codon (ATG, ATA and GTG) and 2 types of termination codon (TAG and TAA) occurred in the *O. ailuri* mt genome; these codons were commonly used in Plagiorchiida trematodes. The result was consistent with most Plagiorchiida trematodes, but different from some Plagiorchiida trematodes, such as *Eurytrema pancreaticum*, *L. longicauda* and *Plagiorchis maculosus* that utilized incomplete stop codon T as termination codon (Chang *et al*., 2016; Suleman *et al*., 2019, 2020). Two NCRs were identified in the *O. ailuri* mt genome, which is consistent with other related Plagiorchiida, such as *Fischoederius elongatus*, *G. crumenifer* and *Paragonimus ohirai* (Yang *et al*., 2015, 2016; Le *et al*., 2020). However, this result is inconsistent with other related Plagiorchiida, such as *Diplodiscus japonicus* and *Tracheophilus cymbius*, in which only 1 NCR has been identified in the mt genome (Li *et al*., 2019; An *et al*., 2022).

The gene *cox*1 was considered to be a useful barcode for metazoans, and widely used for trematode studies (Mioduchowska *et al*., 2018). The mitochondrial genes *cox*1 have also been used to study the population genetic structure of trematode on a local and global scale (Djuikwo-Teukeng *et al*., 2019; Shumenko and Tatonova, 2022; Wang *et al*., 2022). In the present study, the sequence identities and sliding window analysis indicated *cox*1 was the most conserved gene among 12 PCGs in 14 Pronocephalata species; this result was similar to those from a study of *L. longicauda*, *O. sikae*, *N. intestinalis*, *Paramphistomum leydeni* and *T. zarudnyi* (Ma *et al*., 2015, 2016; Suleman *et al*., 2020, 2021; Xu *et al*., 2021).

Gene arrangement in mt genomes provides a source of information for phylogenetic inference (Le *et al*., 2001). Wey-Fabrizius *et al.* (2013) reviewed how mt genome data contribute to the phylogeny debates within Platyzoan, which showed that the gene order of PCGs and rRNA genes within Platyhelminthes were highly conserved with exceptions of partial Monogenea and Seriata trematodes. For majority trematodes, the gene order is *cox*3 > *cyt*b > *nad*4L > *nad*4 > *atp*6 > *nad*2 > *nad*1 > *nad*3 > *cox*1 > *rrn*L > *rrn*S > *cox*2 > *nad*6 > *nad*5 (Wey-Fabrizius *et al*., 2013), which was well supported by the present study. Solà *et al*. (2015) reported the mt genomes and gene order of 2 free-living flatworm species (*Crenobia alpina* and *Obama* sp.), and compared with other relevant helminthes, it was found that the gene order among free-living flatworms differs considerably from the parasitic flatworms. However, the gene order of both free-living and parasitic flatworms was highly conservative with only a few exceptions in each kind of flatworm (Solà *et al*., 2015). The phenomenon was also found in Plagiorchiida trematodes in the present study; 48 Plagiorchiida trematodes available in GenBank shared an identical gene order based on PCGs and rRNA genes.

The gene rearrangement showed 5 types among 48 Plagiorchiida trematodes, and only occurred in transposed tRNAs. This was different from *Schistosoma* trematodes, the gene arrangement of which was radically altered by PCGs (Le *et al*., 2001; Zhang *et al*., 2019). In addition, the rearrangement events mainly occurred in *nad*1-N-P-I-K-*nad*3 and *nad*5-G-E-*cox3* gene blocks of the mt genome in Plagiorchiida. Zhang *et al*. reported that type I (*trn*G-*trn*E) and type II (*trn*E-*trn*G) rearrangements were generated by the gene recombination of ancestral gene rearrangements in Trematoda, and hypothesized that type II evolutionarily pre-dates type I gene rearrangements (Zhang *et al*., 2019). Thus, these gene rearrangements might be an important molecular marker of Plagiorchiida trematode evolution.

Previous research reported the phylogenetic position of *O. sikae* based on the complete mt genome (Ma *et al*., 2016). However, molecular data were rare at that time, and only morphological-based approaches could not fully reflect the phylogenetic relationship within Plagiorchiida trematodes. Thus, the phylogenetic relationships with other Pronocephalata trematodes previously reported were reconstructed based on the concatenation of 12 PCGs. In the phylogenetic tree, most of trematodes were consistent with the current taxonomic status of Plagiorchiida (Olson *et al*., 2003). Notably, except for *T. zarudnyi* (Eucotylidae), 12 Echinostomata trematodes clustered together to form a group. However, *T. zarudnyi* clustered together with Prosthogonimidae flukes, a result consistent with previous phylogenetic assessments (Guo *et al*., 2022). Thus, the current results showed that suborder Echinostomata is polyphyletic. Eucotylidae was considered to belong to Cyclocoeloidea (Strigeida) by Kanev *et al.*, while Olson *et al.* (2003) showed that Eucotylidae should belong to the suborder Xiphidiata as a long-branched clade based on nuclear ribosomal DNA (Kanev *et al*., 2002; Olson *et al*., 2003). This hypothesis was also supported by the present study based on the complete mt genome of *O. ailuri*.

In the branch of Pronocephalata, 3 family trematodes (Paramphistomidae, Gastrothylacidae, Diplodiscidae) clustered together to form a group, and 1 family trematode (Notocotylidae) forming another group. A similar result was also reported in previous studies using the mtDNA genome (Atopkin *et al*., 2021; An *et al*., 2022; Ivashko *et al*., 2022). However, this was not consistent with those of a previous study using ITS*2* for phylogenies, which demonstrated a close genetic affinity of Notocotylidae with Bucephalidae (of the order Strigeidida), rather than with Paramphistomidae and Gastrothylacidae (Xu *et al*., 2021).

In the Notocotylidae branch, *O. ailuri* was closer to *O. sikae* than to *N. intestinalis*, which is consistent with the current taxonomic status of Notocotylidae. These data provide novel and useful genetic markers for studying systematics and population genetics, but only 3 complete mt genomes have been published for Notocotylidae species in GenBank. Therefore, more mt genomes should be sequenced to re-examine the phylogenetic relationships of Pronocephalata and other trematodes.

## Data Availability

All data generated or used during the study appear in the submitted article.

## Supplementary material

For supplementary material accompanying this paper visit https://doi.org/10.1017/S0031182023000379. click here to view supplementary material
