# Supplementary material for: The complete mitochondrial genome of Ogmocotyle ailuri: gene content, composition and rearrangement and phylogenetic implications
Source: Parasitology. 2023 Apr 13;150(8):661–71. doi: 10.1017/S0031182023000379 (PMC10410389; doi:10.1017/S0031182023000379)
Supplement: Supplementary file 1 [file S0031182023000379sup.zip › S0031182023000379sup003.docx]

**Table S1.** Four pairs of primers used for the complete mt genome assembly validation of *Ogmocotyle ailuri*.

| **Regions** | **Primers (5' to 3')** | **Locations** | **Sizes (bp)** |
| --- | --- | --- | --- |
| *cyt*b | F: TTGTTTATCTTTCGTAGGGTC | 629-1141 | ~500 |
|  | R: ATAGAAGTCAAAACTGTAGCA |  |  |
| *nad*2 | F: TCTGGGGTCTTTCTACTGCTT | 4458-5184 | ~720 |
|  | R: GCACGACAAACTGACATTCTC |  |  |
| *rrn*S | F: ATTTACTTAGTTGCCATCTCG | 9347-10128 | ~780 |
|  | R: TTTGTCATAAGCAGCACATAGA |  |  |
| *nad*5 | F: TGGCGATGTATCATTGTTTCTT | 12169-12661 | ~500 |
|  | R: CATACGCAGATAAATACTGTAAGCA |  |  |
