# Supplementary material for: The complete mitochondrial genome of Ogmocotyle ailuri: gene content, composition and rearrangement and phylogenetic implications
Source: Parasitology. 2023 Apr 13;150(8):661–71. doi: 10.1017/S0031182023000379 (PMC10410389; doi:10.1017/S0031182023000379)
Supplement: Supplementary file 1 [file S0031182023000379sup.zip › S0031182023000379sup004.docx]

**Table S2** The information of trematodes reconstructed phylogenetic tree in present study.

| **Suborder** | **Family** | **Species** | **GenBank Number** |
| --- | --- | --- | --- |
| Pronocephalata | Diplodiscidae | *Diplodiscus nigromaculati* | MW698822 |
|  |  | *Diplodiscus japonicus* | OL961442 |
|  |  | *Diplodiscus mehari* | OL961441 |
|  | Paramphistomidae | *Paramphistomum cervi* | NC_023095 |
|  |  | *Calicophoron microbothrioides* | NC_027271 |
|  |  | *Explanatum explanatum* | NC_027958 |
|  |  | *Orthocoelium streptocoelium* | NC_028071 |
|  |  | *Paramphistomum leydeni* | KP341657 |
|  | Gastrothylacidae | *Gastrothylax crumenifer* | NC_027833 |
|  |  | *Fischoederius cobboldi* | NC_030529 |
|  |  | *Fischoederius elongatus* | NC_028001 |
|  | Notocotylidae | *Notocotylus intestinalis* | NC_059797 |
|  |  | *Ogmocotyle sikae* | NC_027112 |
|  |  | *Ogmocotyle ailuri* | OP414758 |
| Xiphidiata | Paragonimidae | *Paragonimus westermani* | MN412706 |
|  |  | *Paragonimus ohirai* | NC_032032 |
|  |  | *Paragonimus heterotremus* | NC_039430 |
|  | Prosthogonimidae | *Prosthogonimus pellucidus* | MZ169556 |
|  |  | *Prosthogonimus cuneatus* | NC_050918 |
|  | Plagiorchiidae | *Glypthelmins quieta* | MZ099629 |
|  |  | *Plagiorchis maculosus* | MK641809 |
|  | Dicrocoeliidae | *Dicrocoelium chinensis* | NC_025279 |
|  |  | *Dicrocoelium dendriticum* | NC_025280 |
|  |  | *Eurytrema pancreaticum* | KP241855 |
|  |  | *Lyperosomum longicauda* | NC_048467 |
|  | Brachycladiidae | *Brachycladium goliath* | KR703278 |
|  | Haploporidae | *Carassotrema koreanum* | NC_065771 |
|  |  | *Parasaccocoelium mugili* | MW846232 |
| Echinostomata | Cyclocoelidae | *Tracheophilus cymbius* | NC_044135 |
|  | Eucotylidae | *Tamerlania zarudnyi* | MW334947 |
|  | Fasciolidae | *Fascioloides magna* | KU060148 |
|  |  | *Fasciola hepatica* | AF216697 |
|  |  | *Fasciolopsis buski* | NC_030528 |
|  |  | *Fasciola jacksoni* | KX787886 |
|  |  | *Fasciola gigantica* | MH621335 |
|  | Echinochasmidae | *Echinochasmus japonicus* | NC_030518 |
|  |  | *Echinostoma hortense* | KR062182 |
|  |  | *Echinostoma miyagawai* | MN116740 |
|  |  | *Echinostoma revolutum* | NC_046395 |
|  |  | *Artyfechinostomum malayanum* | NC_065766 |
|  |  | *Artyfechinostomum sufrartyfex* | NC_037150 |
| Opisthorchiata | Opisthorchiidae | *Clonorchis sinensis* | MT607652 |
|  |  | *Opisthorchis felineus* | NC_011127 |
|  |  | *Opisthorchis viverrine* | JF739555 |
|  |  | *Metorchis orientalis* | NC_028008 |
|  | Heterophyidae | *Cryptocotyle lingua* | NC_063968 |
|  |  | *Haplorchis taichui* | MG972809 |
|  |  | *Metagonimus yokogawai* | NC_023249 |
| Diplostomida | Brachylaimidae | *Postharmostomum commutatum* | NC_010976 |
