# Supplementary figures and images for: The complete mitochondrial genome of Ogmocotyle ailuri: gene content, composition and rearrangement and phylogenetic implications
Source: Parasitology. 2023 Apr 13;150(8):661–71. doi: 10.1017/S0031182023000379 (PMC10410389; doi:10.1017/S0031182023000379)

## Slide 1
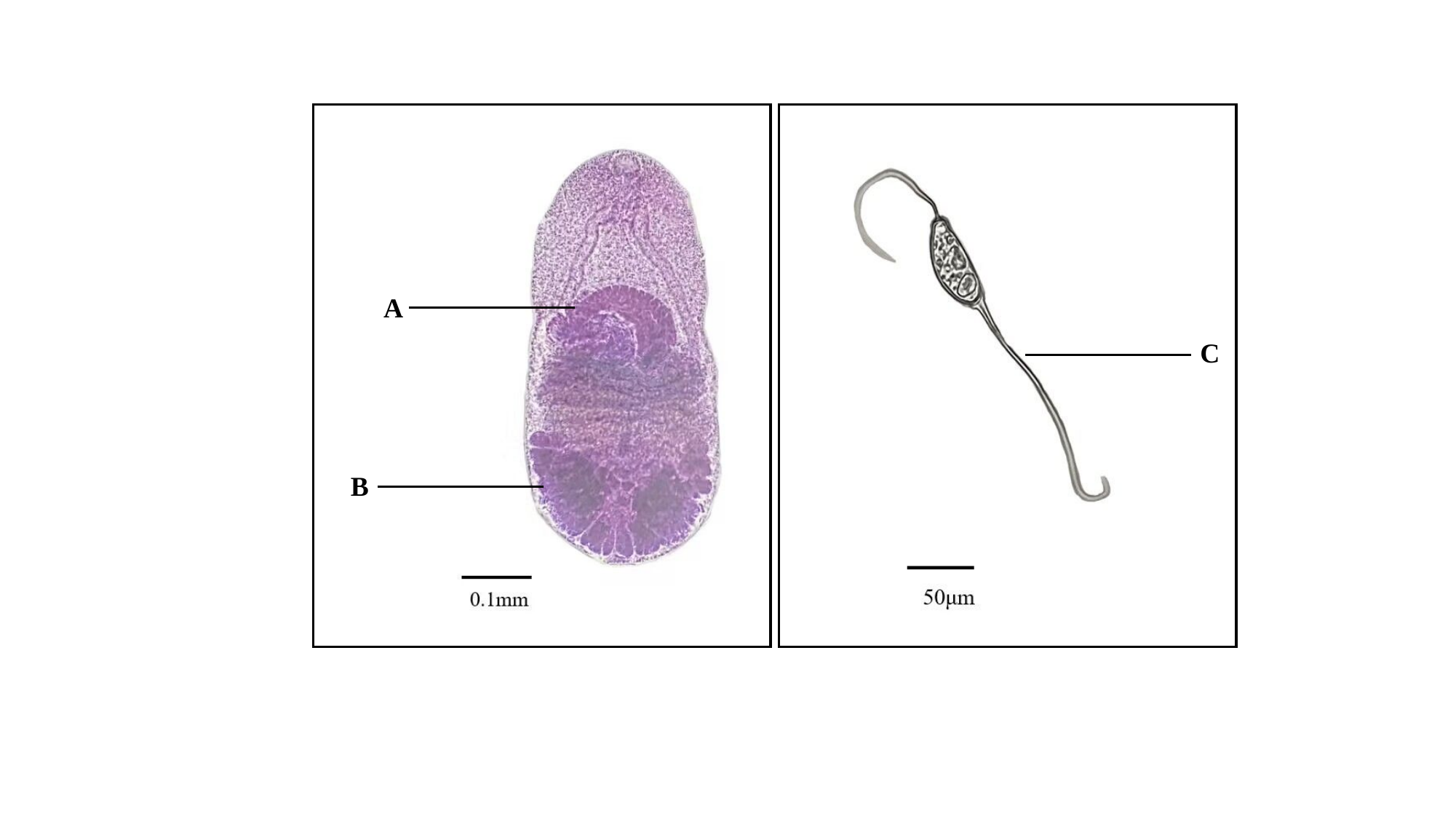

A
C
B

Supplement: Supplementary file 1 [file S0031182023000379sup.zip › S0031182023000379sup001.pptx]

## Slide 1
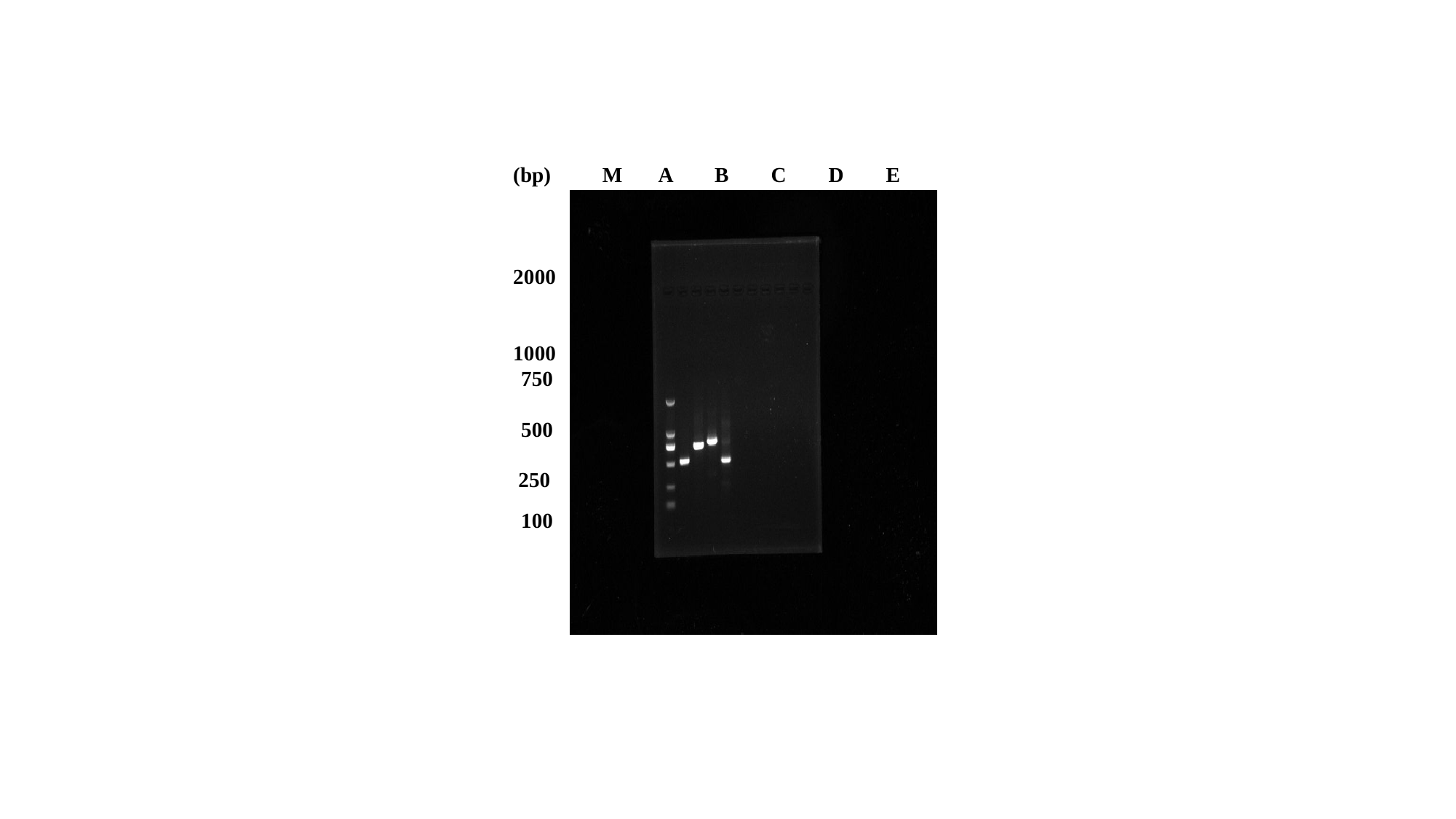

M A B C D E
(bp)
2000
1000
750
500
 250
100

Supplement: Supplementary file 1 [file S0031182023000379sup.zip › S0031182023000379sup002.pptx]
